# Analysis and prediction of the coronavirus disease epidemic in China based on an individual-based model

**DOI:** 10.1038/s41598-020-76969-4

**Published:** 2020-12-17

**Authors:** Zuiyuan Guo, Dan Xiao

**Affiliations:** 1 Department of Disease Control, Center for Disease Control and Prevention in Northern Theater Command, Shenyang, China; 2 China National Clinical Research Center for Neurological Diseases, Beijing Tiantan Hospital, No. 119, South 4th Ring Road West, Fengtai District, Beijing, China

**Keywords:** Infectious diseases, Mathematics and computing

## Abstract

We established a stochastic individual-based model and simulated the whole process of occurrence, development, and control of the coronavirus disease epidemic and the infectors and patients leaving Hubei Province before the traffic was closed in China. Additionally, the basic reproduction number (*R*_0_) and number of infectors and patients who left Hubei were estimated using the coordinate descent algorithm. The median *R*_0_ at the initial stage of the epidemic was 4.97 (95% confidence interval [CI] 4.82–5.17). Before the traffic lockdown was implemented in Hubei, 2000 (95% CI 1982–2030) infectors and patients had left Hubei and traveled throughout the country. The model estimated that if the government had taken prevention and control measures 1 day later, the cumulative number of laboratory-confirmed patients in the whole country would have increased by 32.1%. If the lockdown of Hubei was imposed 1 day in advance, the cumulative number of laboratory-confirmed patients in other provinces would have decreased by 7.7%. The stochastic model could fit the officially issued data well and simulate the evolution process of the epidemic. The intervention measurements nationwide have effectively curbed the human-to-human transmission of severe acute respiratory syndrome coronavirus 2.

## Introduction

Since December 8, 2019, patients with fever, cough, myalgia, and fatigue have successively emerged in Wuhan, Hubei Province, Central China^1–3^. They had pneumonia with abnormal findings on chest computed tomography^2,3^. The National Health Commission (NHC), Wuhan Health Commission, and Chinese Center for Disease Control and Prevention (CDC) launched an investigation into the epidemic and found that several patients had a history of exposure to the Huanan Seafood Market, which was announced on December 31, 2019^1^. On January 1, 2020, this market was closed^1^. On January 8, the CDC officially announced that a novel coronavirus (severe acute respiratory syndrome coronavirus 2 [SARS-CoV-2]) was the causative pathogen for the outbreak^1,4,5^. On January 23, to prevent the epidemic from spreading to the whole country, the Wuhan government ordered the closure of roads and railways and cancellation of flights to other places and prohibited all people from leaving Wuhan^6^. One day later, the traffic to and from Hubei were blocked. On January 25, the leading national group for epidemic response was established^7^. Based on the experience of severe acute respiratory syndrome (SARS) prevention and control, the epidemic prevention and control measures were rapidly implemented nationwide under the unified leadership of the central government.

After the outbreak of the coronavirus disease (COVID-19) epidemic, epidemiologists have used various mathematical models to conduct epidemiological studies. For example, Wu et al. established the Susceptible-Exposed-Infectious-Recovered (SEIR) model, with an estimated basic reproduction number (*R*_0_, the expected number of secondary cases derived from a typical infection entering a completely susceptible population during its infectious period) of 2.68^8^. Meanwhile, Du et al. estimated that there would be 12,400 infectors in Wuhan by January 22 using a simple model of exponential growth coupled with a stochastic model^9^. These studies have played a positive role in people’s understanding of epidemiological characteristics. However, most studies have only calculated the *R*_0_ at the initial stage of the epidemic, regardless of whether this value changed with the progress of intervention measures^8–11^. Moreover, several models do not perform quantitative analysis on the association between the number of new infectors per day and the number of laboratory-confirmed patients or the impact of Hubei’s lockdown and the government’s intervention measures on the epidemic nationwide.

In this study, an individual-based model was established based on the concept of randomization, which reproduced the whole process of occurrence, development, and control of the epidemic. This model can overcome the limitations of the traditional SEIR model and has unique advantages. First, the parameters of the stochastic model for each infector are randomly assigned according to certain probability distribution rules. Second, the stochastic model can flexibly set the activities of infectors, including randomly selecting some infectors and patients to travel to other provinces before Hubei was locked down. Third, the stochastic model can also simulate aggregated epidemics and super-disseminators. For example, when the model is run on a computer, some infectors can occasionally spread with a high density in a short period or spread in large quantities to susceptible persons. Finally, the randomization model can calculate the fluctuation ranges of the numbers of new and accumulative patients through repeated calculations, helping people predict various possibilities of epidemic development. The various features show that the stochastic model can reproduce the reality and predict the developmental trend of the epidemic more accurately through more flexible details than the other models. It has scientific reference value for people to assess the epidemic situation and evaluate the effects of intervention measures.

## Results

### Transmission chain

**Figure 1:**
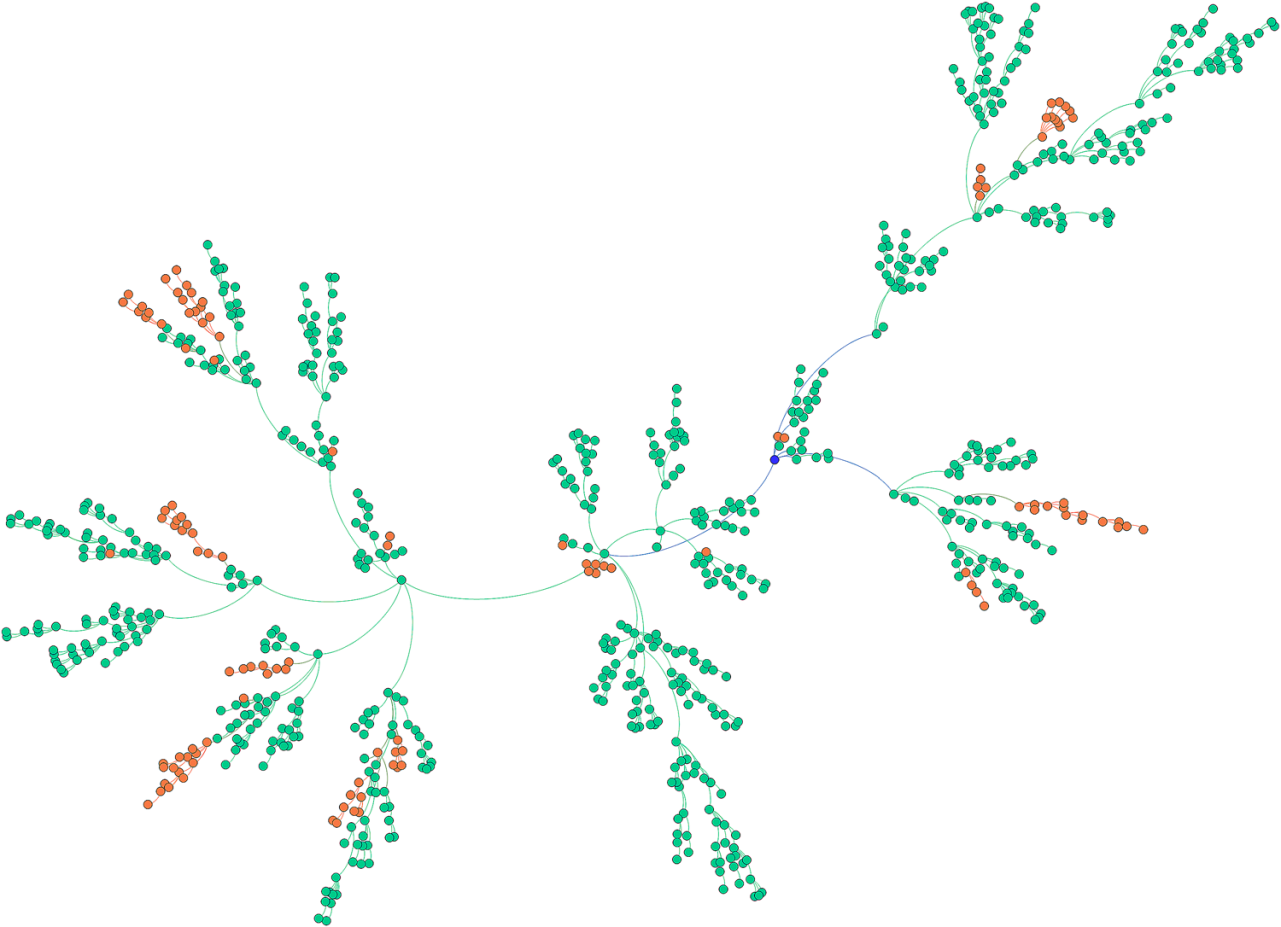
Transmission chain of 802 infectors caused by one infection source. The green circles represent the infectors in Hubei; the red circles represent the infectors in other provinces throughout China; the blue circle represents the first infectious source in the transmission chain; the connection lines represent the transmission associations.

We randomly selected a patient with an exposure history to the Huanan Seafood Market. We found that 802 individuals were infected due to human-to-human transmission of SARS-CoV-2 and visualized the transmission network (Fig. 1). Among them, the most contagious person could infect nine susceptible individuals and was considered a super-disseminator in the model.

### Average instantaneous basic reproduction number at timepoint *t*

**Figure 2:**
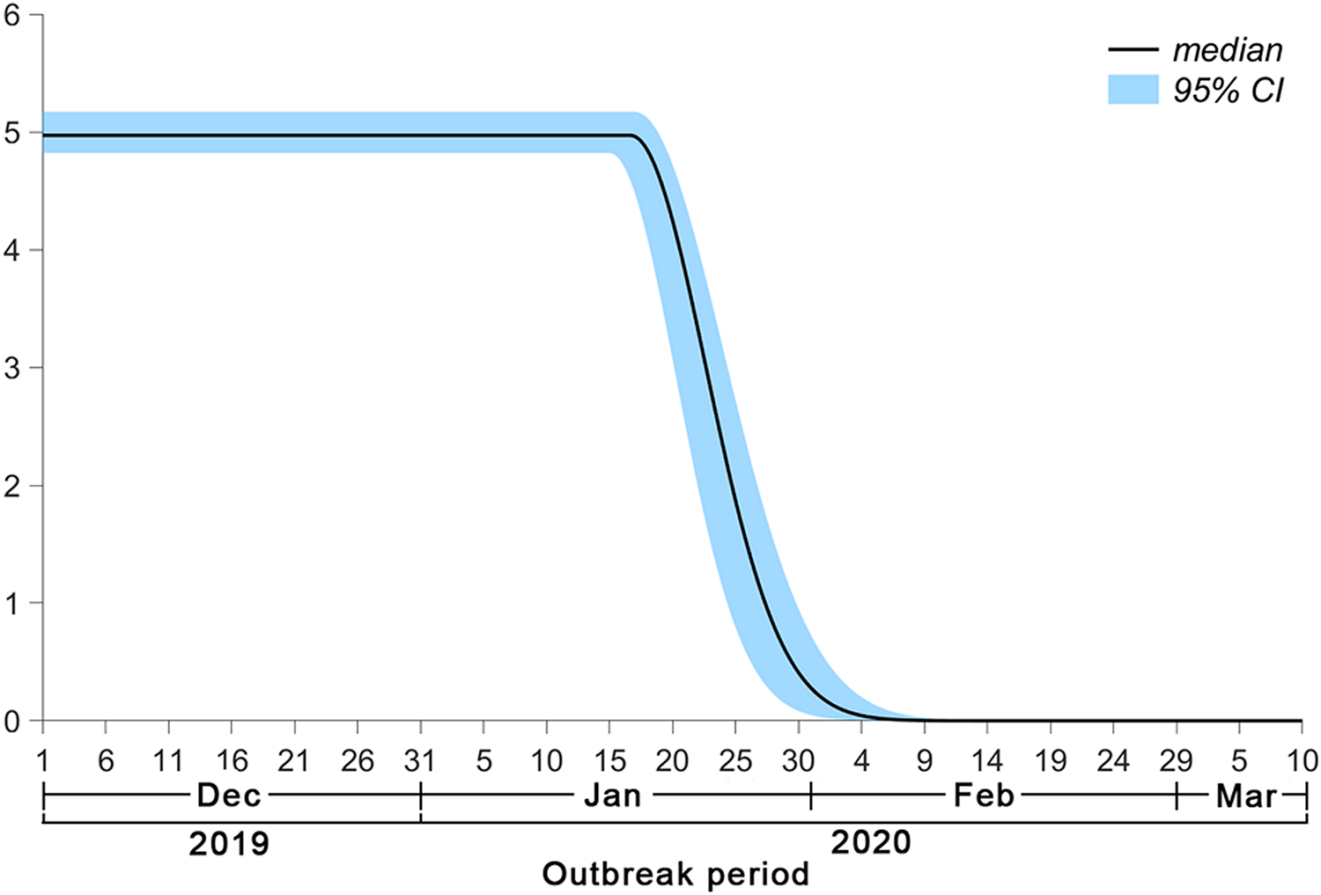
Temporal distribution of the *R*_0_(*t*). The solid line represents the median value of *R*_0_(*t*), and the blue area represents the 95% confidence interval.

After testing all possible the average instantaneous basic reproduction number at timepoint *t* (*R*_0_(*t*)) functions, the minimum Akaike information criterion (AIC) can be obtained when the *R*_0_(*t*) remains constant at first and subsequently decreases exponentially. Furthermore, the instantaneous basic reproduction number of Hubei and other provinces (*R*_0*h*_(*t*) and *R*_0*n*_(*t*), respectively) were equal in the expression and parameters of the function based on the results of the calculation. Figure 2 shows the changes in *R*_0_(*t*) over time. From early December 2019 to January 16, 2020, the *R*_0_(*t*) remained constant with a median of 4.97 (95% confidence interval [CI] 4.82–5.17). Since then, the *R*_0_(*t*) has shown a rapid downward trend, decreasing to less than 1 by January 27, 2020.

### Numbers of new laboratory-confirmed patients and new infectors

**Figure 3:**
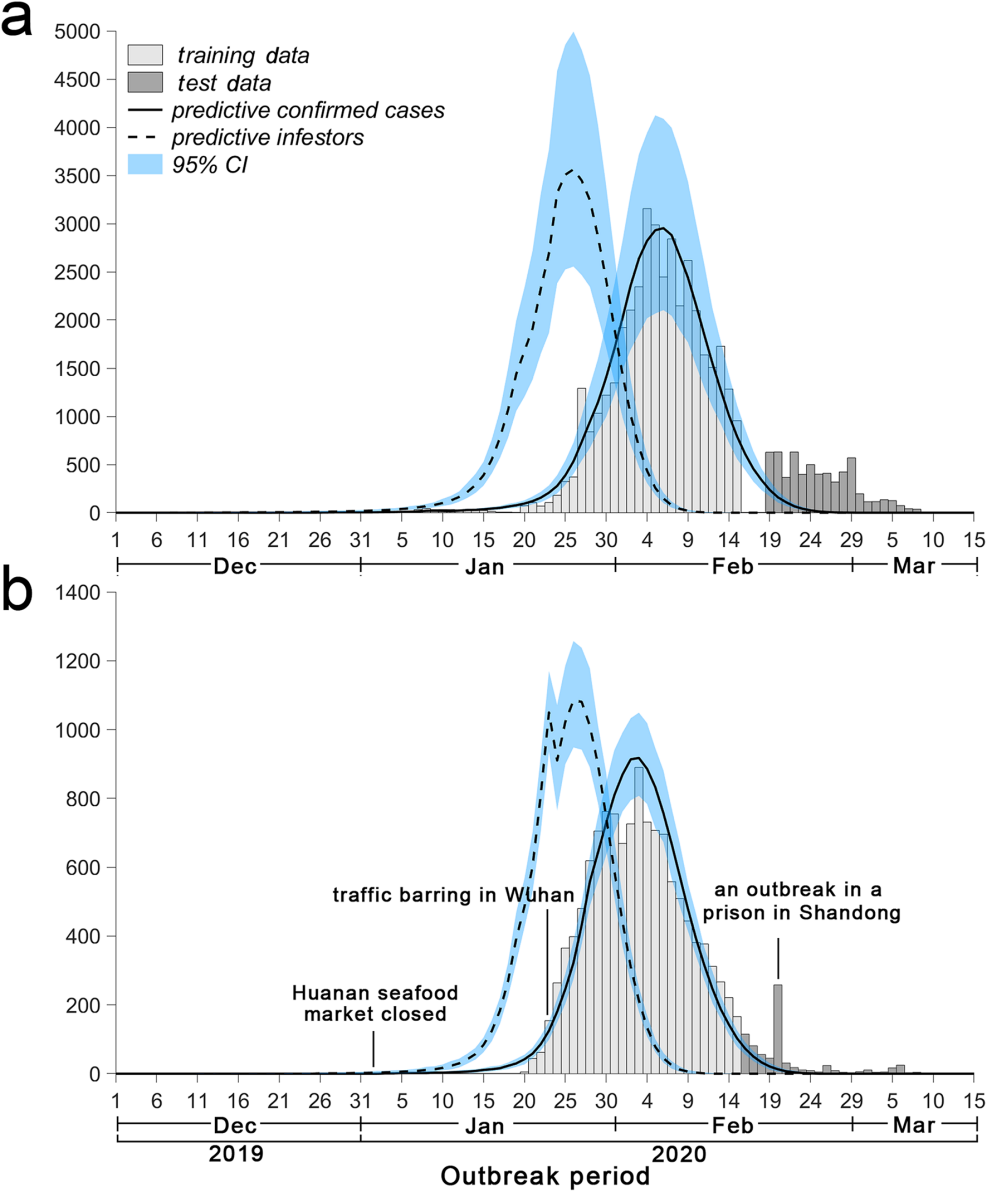
Numbers of new laboratory-confirmed patients and new infectors estimated by the model. Histogram (**a**) represents the number of new laboratory-confirmed patients in Hubei issued by the government, and the solid and dashed lines represent the numbers of new laboratory-confirmed patients and new infectors in Hubei estimated by the model, respectively. From February 16 to 18, the number of new laboratory-confirmed patients in Hubei could not be obtained because the number of new laboratory-confirmed patients and clinically diagnosed patients was simultaneously released by the HHC. Histogram (**b**) represents the number of new laboratory-confirmed patients in other provinces issued by the government. The abrupt rise in this number on February 20 was owing to a clustering epidemic in a prison in Shandong Province^12^. The solid and dashed lines represent the number of new laboratory-confirmed patients and number of new infectors in other provinces estimated by the model, respectively.

Figure 3a shows that the number of new laboratory-confirmed patients in Hubei predicted by the model peaked on February 6, with an average number of 2953 (95% CI 2103–4088), while the number of new infectors peaked 11 days in advance and was 3563 (95% CI 2554–4992). Figure 3b shows that the number of new laboratory-confirmed patients in other provinces peaked on February 3, with an average number of 918 (95% CI 808–1049), while the number of new infectors peaked 11 days in advance and was 1084 (95% CI 947–1248). On January 24, as the province of Hubei was under lockdown, the number of new infectors exported to other provinces decreased.

### Cumulative number of laboratory-confirmed patients

**Figure 4:**
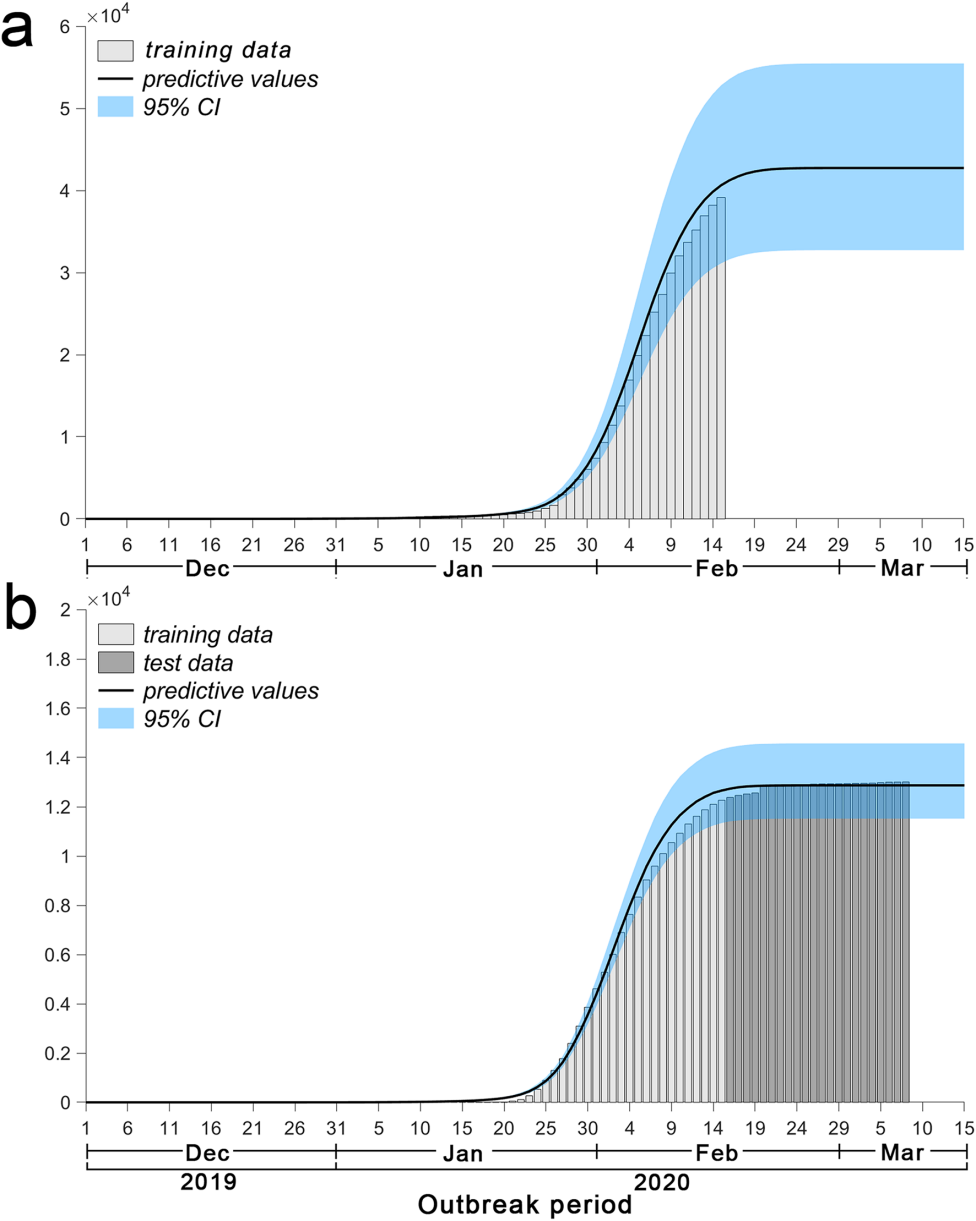
Cumulative number of new laboratory-confirmed patients estimated by the model. (**a**) Represents the cumulative number of new laboratory-confirmed patients in Hubei estimated by the model. (**b**) Represents the cumulative number of new laboratory-confirmed patients in other provinces estimated by the model.

Figure 4a shows that the cumulative number of laboratory-confirmed patients in Hubei predicted by the model reached 42,739 on March 15 (95% CI 32,734–55,472). Figure 4b shows that the cumulative number of laboratory-confirmed patients in other provinces predicted by the model reached 12,870 on March 15 (95% CI 11,520–14,572).

### Numbers of unidentified patients and unidentified infectors

**Figure 5:**
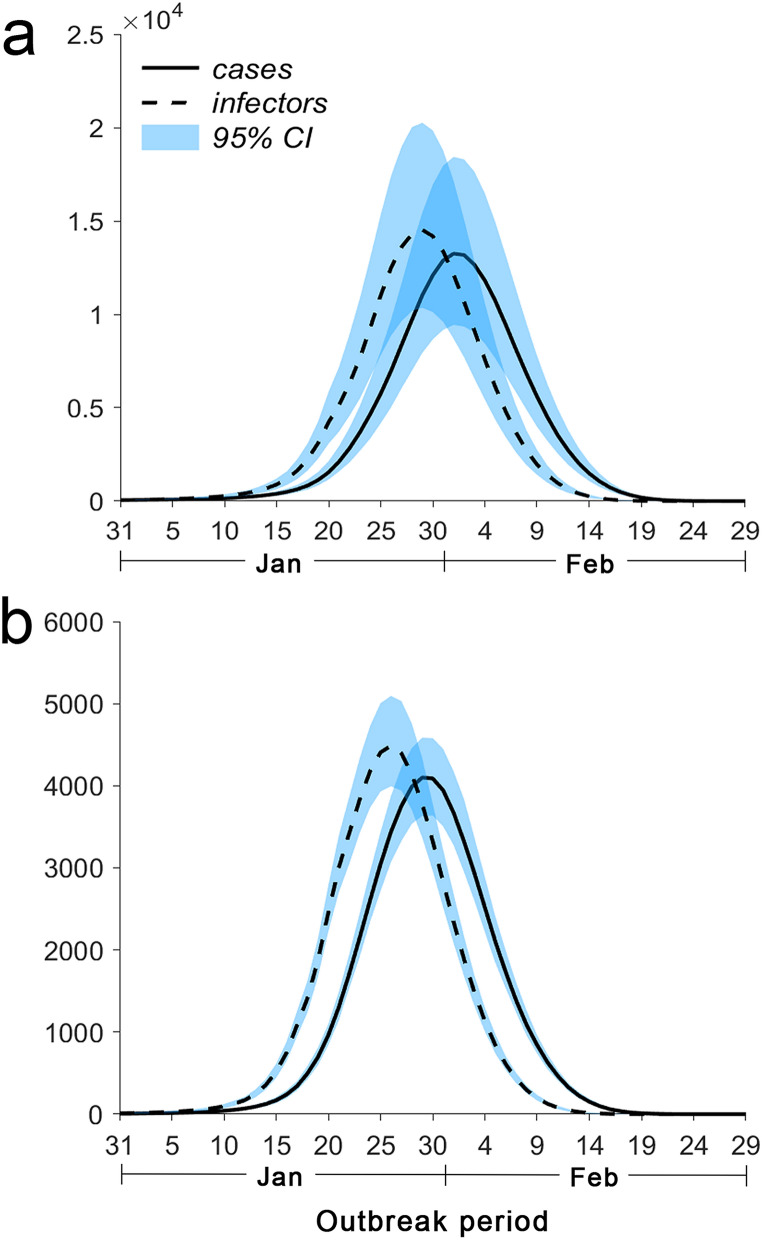
Unidentified patients and infectors estimated by the model. (**a**) The solid and dashed lines represent the numbers of unidentified patients and unidentified infectors in Hubei estimated by the model, respectively. (**b**) The solid and dashed lines represent the numbers of unidentified patients and unidentified infectors in other provinces estimated by the model, respectively.

Figure 5a shows that the number of unidentified patients in Hubei peaked on February 1, with a median of 13,252 (95% CI 9434–18,434). The number of unidentified infectors in Hubei peaked on January 29, with a median of 14,524 (95% CI 13,218–16,058). Figure 5b shows that the number of unidentified patients in other provinces peaked on January 29, with a median of 4100 (95% CI 3634–4586). The number of unidentified infectors in other provinces peaked on January 26, with a median of 4488 (95% CI 4274–4687).

### Impact of delaying intervention measures and lockdown of Hubei in advance on the epidemic developmental trend

**Figure 6:**
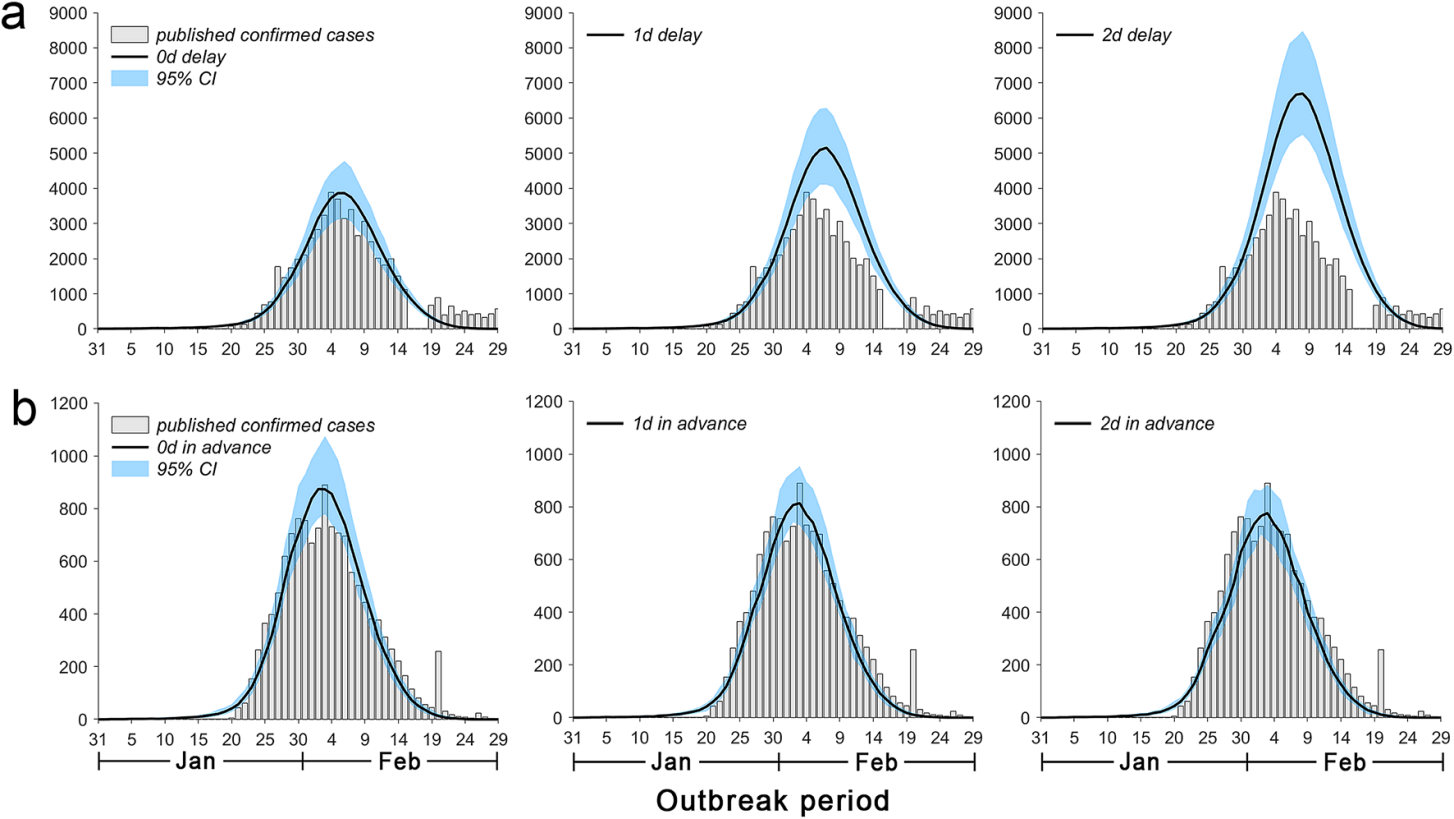
Impact of delayed intervention measures and lockdown of Hubei in advance on the epidemic developmental trend. (**a**) Represents the impact of delayed intervention measures on the number of new laboratory-confirmed patients nationwide. The histogram represents the number of new laboratory-confirmed patients nationwide issued by the government, and the solid line indicates the number of new laboratory-confirmed patients nationwide predicted by the model. (**b**) Represents the impact of Hubei lockdown in advance on the number of new laboratory-confirmed patients in other provinces. The histogram represents the number of new laboratory-confirmed patients in other provinces reported by the government, and the solid line represents the number of new laboratory-confirmed patients in other provinces predicted by the model.

Figure 6a shows the impact of delaying intervention measures on the epidemic nationwide. When the intervention measures were delayed for 1 and 2 days, the peak in the number of new laboratory-confirmed patients in the whole country was postponed by 1 and 2 days, respectively, and the cumulative number increased by 32.1% and 73.4% on March 15, respectively. Figure 6b shows that when the lockdown in Hubei was implemented 1 and 2 days in advance, the peak in the number of new laboratory-confirmed patients in other provinces occurred 1 day in advance and the cumulative number decreased by 7.7% and 11.6% on March 15, respectively.

### Sensitivity analysis

We obtained 500 samples from a uniform distribution for each parameter ($\alpha$, $\beta$, and $\tau$ of *R*_0_(*t*)) range, and the Partial rank correlation coefficients (PRCCs) for the three indexes were 0.87, 0.57, and − 0.18, respectively. A value greater than 0 indicates a positive correlation, while a value less than 0 indicates a negative correlation. Values near − 1 or + 1 indicate that the parameter has a strong impact on the output, whereas values closer to 0 indicate less effect on the output result.

## Discussion

First, we have discussed the impact of *R*_0_(*t*), new infectors, unidentified patients and infectors, intervention measures, and the significance of this study for epidemiology and public health. Then, we have discussed the limitations of this study.

*R*_0*h*_(*t*) and *R*_0*n*_(*t*) were equal, suggesting that the intensity of the virus transmission and implementation of prevention and control measures in Hubei and other provinces were comparable. In this study, the *R*_0_(*t*) at the initial stage of the epidemic was 4.97, which was not only higher than the value reported in previous studies (2.2^1^ and 2.46^8^) but also higher than the value reported during the SARS pandemic (2.87^13^). This can reasonably explain why the growth rate and cumulative number of patients during the COVID-19 pandemic were higher than those of during the SARS pandemic, although the Chinese government has implemented similar intervention measures as those for SARS. This study found that the *R*_0_(*t*) remained high until January 16, which led to a rapid increase in the number of patients nationwide. After January 16, the *R*_0_(*t*) decreased rapidly. This timepoint is consistent with the time that strict exit screening measures were activated in Wuhan and when people with body temperature ≥ 37.3 °C were restricted from leaving Wuhan^1^. The research results were verified by facts. The rapid decrease in the *R*_0_(*t*) was the main reason for the significant decrease in the number of new laboratory-confirmed patients nationwide.

Based on Fig. 3, the changes in new infectors were similar to those in the number of new laboratory-confirmed patients, both initially increasing and subsequently decreasing. This is because patients can infect susceptible persons only in the period after disease onset and before isolation treatment. Therefore, the number of unidentified patients and *R*_0_(*t*) influence the number of new infectors. After February 19, the number of laboratory-confirmed patients reported by the Hubei Provincial Health Commission (HHC) were significantly higher than the number predicted by the model. This was probably because from February 17, Wuhan launched a 3-day dragnet investigation of close contacts with confirmed patients, suspected patients, and patients with fever in whom the presence of COVID-19 could not be ruled out^14^. In addition, since February 18, a comprehensive investigation and verification of patients with fever were also conducted in Hubei^15^. However, most patients with mild illness and asymptomatic infections do not seek medical attention and are diagnosed under normal conditions; therefore, the government’s active investigation was unable not meet the first premise of the model.

Determining the numbers of unidentified infectors and patients helps the government formulate effective prevention and control plans. However, these numbers cannot be directly obtained in practice. We performed a analysis of unidentified infectors and patients’ quantitative association in Fig. 5 and found that the number of unidentified infectors changes before that of unidentified patients changes, suggesting that onset of disease occurs in the unidentified infectors after a period of time. The peak timepoints of unidentified infectors and patients are 4–8 days earlier than the peak timepoint of new laboratory-confirmed patients. Thus, the peak time points of unidentified infectors and patients can be predicted based on the peak time point of new laboratory-confirmed patients.

The earlier the intervention measures were implemented, the easier it was to control the epidemic (Fig. 6a). To prevent the epidemic from spreading to the whole country, the government blocked the traffic to and from Hubei. As shown in Fig. 6b, the lockdown of Hubei helped contain the epidemic throughout the country. Although this measure increased the number of patients in Hubei, it controlled the epidemic in the local area, reduced the risk of spreading the epidemic to the whole country, and significantly reduced the cost of fighting the epidemic, thus allowing the country to concentrate national medical forces to support Wuhan and other cities in Hubei.

Compared with traditional dynamic models, the individual-based model incorporates the idea of artificial intelligence. It studies the interactions among the infection source, transmission route, and susceptible population from the individual level, which is a powerful supplement to the SEIR model. The model can set the behavior of COVID-19 patients, asymptomatic individuals, and the government more flexibly to make the model closer to the actual situation and improve the ability to predict the epidemic scenario. Additionally, the individual-based model can be applied to epidemiological studies of other infectious diseases. For example, we used the model to simulate adenovirus type 7 in the military^16^, and the results showed that the model has good applicability for quantitative analysis of the epidemiological characteristics of cluster outbreaks.

This study has two limitations. First, some patients who were asymptomatic and those with mild symptoms who did not seek medical attention were not included in the list of laboratory-confirmed patients; therefore, the number of laboratory-confirmed patients issued by the state is less than the actual number, which would lead to the underestimation of *R*_0_(*t*). Furthermore, the time-varying reporting ratio affected by low testing accuracy and limited knowledge on SARS-CoV-2 at the early stage of the epidemic influenced the evaluation of *R*_0_(*t*). However, correcting the official data is beyond our jurisdiction and ability, and the official data we acquired are currently the most reliable. Second, although asymptomatic patients are not the main source of infection, some are considered infectious. Because the country lacks accurate statistics for asymptomatic patients, we cannot estimate their scale in this study. Hence, a new model investigating this theoretically needs to be established. These limitations may lead to deviations in the model’s estimation of the number of patients and infectors.

The individual-based model fits well with the official data and is consistent with the facts, suggesting that the model can reasonably reflect the developmental trend of the epidemic and provides a good reference for epidemic analysis in other countries and regions.

## Methods

### Data

From January 20, 2020, the NHC and HHC reported the number of new laboratory-confirmed patients across the country and in Hubei, respectively, on a daily basis^17,18^. According to the Diagnosis and Treatment Plan for Novel Coronavirus Pneumonia (5th Edition)^19^, the number of laboratory-confirmed patients in Hubei was no longer reported separately by the HHC from February 16. Although the 6th edition of the plan issued on February 18 requires the release of the number of laboratory-confirmed patients separately^20^, we were unable to obtain these data in Hubei from February 16 to February 18. Hence, we used the number reported before February 15 as the training data in Hubei and other provinces. The baseline parameters can be obtained from the literature published by the CDC^1^, as shown in Table 1.

**Table 1:** Values of model parameters.

Parameters	Distribution characteristics	Numerical values	Sources
Incubation period	Logarithmic normal distribution	$\mu = 5.2$ $\sigma = 0.87$	^1^
Infection period (time from disease onset to seeking medical attention)	Weibull distribution	Before January 1, 2020 $\mu = 5.8$ $\sigma = 0.87$ After January 1, 2020 $\mu = 4.6$ $\sigma = 0.26$	^1^
*R*_0_(*t*)	$\alpha\quad 0 \le t < \tau$ $\alpha {\text{e}}^{{ - \beta (t - \tau )^{2} }} \quad t \ge \tau$	$\alpha = 4.97$ (95% CI 4.82–5.17) $\beta = 0.014$ (95% CI 0.010–0.018) $\tau = 47.66$ (95% CI 46.00–48.00)	Model estimation
Number of infectors and patients who have left Wuhan	constant *m*	2000 (95% CI 1982–2030)	Model estimation
Time from treatment to definitive diagnosis	Uniform distribution	$\mu = 1.5$ $\sigma = 0.08$	Model assumption

### Model establishment

#### Assumptions for model establishment

We set some preconditions for the model. First, patients were considered infectious only after disease onset, and asymptomatic infectors were not considered infection sources. We defined the infection source based on the 6th edition of the diagnosis and treatment plan^20^ and referred to the infectivity characteristics of patients with SARS^21^. Moreover, only patients seeking medical attention could be diagnosed, while those with asymptomatic infection not seeking medical attention were excluded from the laboratory-confirmed patients included in the model. Second, since COVID-19 is a novel infectious disease and people have no immunity against this disease, all close contacts are considered susceptible. Third, the number of susceptible persons infected by one infector followed a Poisson distribution with *R*_0_ as the mean value. Fourth, Hubei and other provinces had different *R*_0_(*t*) values, possibly because of some differences in time and effect in the implementation of intervention measures in Hubei and other provinces. For example, Hubei faced a serious shortage of medical resources compared with other provinces. We used *R*_0*h*_(*t*) and *R*_0*n*_(*t*) to represent the instantaneous basic reproduction number of Hubei and other provinces, separately.

#### Establishment of the R_0_(t)

Based on the temporal distribution of new laboratory-confirmed patients, we predicted that before the intervention measures were initiated, the virus would continuously spread along with daily contact among people, and *R*_0_(*t*) would continue to remain high during this period. After the intervention measures were initiated, the effective contact frequency among people would be significantly reduced and the infection period of patients would be significantly shortened due to active screening; therefore, *R*_0_(*t*) in this stage would show a downward trend.

Accordingly, it is necessary to establish *R*_0_(*t*) and conduct parameter estimation. The function of *R*_0_(*t*) needs to fulfill two criteria: it must be sufficiently smooth and concise. To select functions, the AIC was implemented. First, the time distribution scatter plot of the official daily number of confirmed cases in Hubei and other provinces were separately plotted. Then, the most suitable function was selected from many common functions, such as linear, quadratic, exponential, Gaussian, Gamma, and logarithmic Gaussian, according to the scatter plot characteristics. The coordinate descent algorithm was used on the selected function for parameter estimation. Finally, the function with the minimum AIC was used as the optimal estimate for *R*_0_(*t*).

#### Establishment of the model according to different developmental stages of the epidemic

In the process of model establishment, we divided the epidemic into three stages according to its occurrence, development, and control processes and designed a computer program according to the characteristics of different stages:

The first stage was the emission period of the epidemic from early December 2019 to January 1, 2020, when the Huanan Seafood Market was closed. The main epidemic features at this stage were as follows. First, animal infection sources in the market continued to spread the virus to humans, leading to the successive appearance of patients with pneumonia^22^. Second, the infected patients were also new infection sources, spreading the virus to other close contacts. In the model, 50 patients were identified as human infection sources at the early stage of the epidemic with an exposure history to the market and 27 patients had unknown causes before the closure of this market, based on CDC findings^1^. Time of infection, time of seeking medical attention, time of transmission to other susceptible persons, their *R*_0_, and other information were calculated and stored in a matrix.

The second stage was the development period of the epidemic from January 1, 2020 to January 25, 2020, when the Chinese government created a leading group to respond to the epidemic and coordinate national epidemic prevention and control measures. The first characteristic of this stage was that people did not adapt to effective protection, resulting in the transmission of the virus among people and, thus, leading to the spread of the epidemic. The second characteristic was that the outbreak started during the Spring Festival travel rush in China, and some infectors left Hubei and traveled to all regions of the country and even abroad. Therefore, we randomly selected some of the infectors and patients as infection sources who arrived at other provinces before Hubei was locked down entirely on January 24. Since then, all the new infectors throughout the country, except those in Hubei, were infected by these infection sources.

The third stage was the control period of the epidemic, starting from January 25, 2020. The government has strictly implemented a series of powerful measures that have gradually curbed the spread of the epidemic^5^. In the different developmental stages of the epidemic, we assigned the *R*_0_ values to patients according to their timepoints of disease onset; hence, the epidemic developmental trend changed with the *R*_0_(*t*).

#### Coordinate descent algorithm

We used the coordinate descent algorithm, an efficient numerical optimization method for solving function extreme values through continuous iteration in machine learning to obtain the parameters^23^. In each round of calculation, we adjusted the size of the parameters to be solved to minimize the objective function. After several iterations, the optimal combination of parameter values with the minimum objective function value was obtained. We took the quadratic sum function of the difference between the number of daily new laboratory-confirmed patients estimated by the model and the corresponding data issued by the government as the objective function. We used the four parameters in the model ($\alpha$, $\beta$, and $\tau$ of the *R*_0_(*t*) and the number of patients and infectors who left Hubei before the locked down, namely, *m*) as the parameters to be estimated (Table 1). Subsequently, we conducted a numerical calculation using the coordinate descent algorithm to obtain the values of the parameters when the objective function reached the minimum value.

#### CIs and prediction intervals

Because the results of the stochastic simulation of the epidemic were different in each round, fixed values of the necessary parameters could not be obtained when the coordinate descent algorithm was used to estimate the values. Therefore, we used the bootstrap method, a statistical method commonly used in research^24^ for interval estimation in nonparametric statistics to obtain the *N* calculation results for each parameter after repeating the calculations *N* times. Subsequently, the CI of each parameter were obtained. Then, we entered the median of each parameter into the model to obtain the prediction interval of the model through cycle computing. The cycle computing iterations of the parameters’ CIs and model prediction intervals were 500 times.

### Sensitivity analysis

PRCC combined with Latin hypercube sampling was used for the sensitivity analysis to evaluate the influence of the three parameters $\alpha$, $\beta$, and $\tau$ of *R*_0_(*t*) on the model output (the total number of accumulative laboratory-confirmed patients nationwide until March 10, 2020). A standard correlation coefficient, *ρ*, was calculated for the parameter and model output^25,26^. Details of the coordinate descent algorithm and sensitivity analysis are shown in Supplementary Material.

## Supplementary information

Supplementary Information.

## Data Availability

The datasets generated or analyzed during the current study are available in the supplemental materials.
