# Supplementary material for: Analysis and prediction of the coronavirus disease epidemic in China based on an individual-based model
Source: Sci Rep. 2020 Dec 17;10:22123. doi: 10.1038/s41598-020-76969-4 (PMC7747602; doi:10.1038/s41598-020-76969-4)
Supplement: Supplementary file 1 — Supplementary Information. [file 41598_2020_76969_MOESM1_ESM.docx]

**Supplemental material for the analysis and prediction of the coronavirus disease epidemic in China based on an individual-based model**

Zuiyuan Guo, Dan Xiao

**Coordinate descent algorithm**

The coordinate descent algorithm is an efficient optimization method of solving extreme values in machine learning [1]. Let *S* be the sum of squares function for the estimation of model parameters using the least squares method and $\alpha$ represent parameters that require estimation. All the variables were held except for some $\alpha_{i}$ fixed and *S*, which was optimized with respect to adjusting the parameter $\alpha_{i}$ in its variable range. Subsequently, we re-optimized the variables in the order$\alpha_{1}$, $\alpha_{2}$, …, $\alpha_{m}$, $\alpha_{1}$, $\alpha_{2}$, … until all the parameters were invariant. Here, *m* represented the number of parameters. When *S* reached its minimum, we then successively estimated the three parameters for $\alpha$, $\beta$, and $\tau$ of $R_{0}$. We used $R(t)$ and $U(t)$ to represent the number of new laboratory-confirmed cases in Hubei Province and other provinces predicted by the model on the *t*th day, respectively. Values of $Data^{hubei}(t)$ and $Data^{others}(t)$ represent new laboratory-confirmed cases in Hubei Province and other provinces issued by the government on the *t*th day, respectively, which were obtained from Table S1. Equation 1 represents objective function $S$ comprising fitting results of Hubei Province and other provinces:

$S(\alpha_{1},\alpha_{2},\cdots,\alpha_{m})=\sum_{t=1}^{77} \left[R(t)-Data^{hubei}(t)\right]^{2}+\left[U(t)-Data^{others}(t)\right]^{2}$ (1)

**Sensitivity analyses**

We performed sensitivity analyses of four significant parameters to assess the impact on the attack rate. Partial rank correlation coefficients (PRCCs) and Latin hypercube sampling were used when performing sensitivity analyses. PRCC-LHS is an efficient and reliable sampling-based sensitivity analysis method that provides a measure of monotonicity between a set of parameters and the model output after the removal of the linear effects of all parameters except the parameter of interest [2,3]. Each parameter interval (from 0.5 to 1.5 times the average value of the parameters) was divided into *N* smaller and equal intervals, and one sample was selected randomly from each interval [2,3]. A standard coefficient denoting the correlation between the parameter and the model output was calculated.

**Table S1. Number of new laboratory-confirmed cases per day in Hubei Province and other provinces issued by the government**

| **Date** | **Hubei**  **Province** | **Other**  **Provinces** |
| --- | --- | --- |
| 1-Dec-19 | 0 | 0 |
| 2-Dec-19 | 0 | 0 |
| 3-Dec-19 | 0 | 0 |
| 4-Dec-19 | 0 | 0 |
| 5-Dec-19 | 0 | 0 |
| 6-Dec-19 | 0 | 0 |
| 7-Dec-19 | 0 | 0 |
| 8-Dec-19 | 1 | 0 |
| 9-Dec-19 | 0 | 0 |
| 10-Dec-19 | 1 | 0 |
| 11-Dec-19 | 0 | 0 |
| 12-Dec-19 | 0 | 0 |
| 13-Dec-19 | 1 | 0 |
| 14-Dec-19 | 0 | 0 |
| 15-Dec-19 | 2 | 0 |
| 16-Dec-19 | 1 | 0 |
| 17-Dec-19 | 1 | 0 |
| 18-Dec-19 | 0 | 0 |
| 19-Dec-19 | 2 | 0 |
| 20-Dec-19 | 4 | 0 |
| 21-Dec-19 | 4 | 0 |
| 22-Dec-19 | 3 | 0 |
| 23-Dec-19 | 4 | 0 |
| 24-Dec-19 | 0 | 0 |
| 25-Dec-19 | 4 | 0 |
| 26-Dec-19 | 1 | 0 |
| 27-Dec-19 | 3 | 0 |
| 28-Dec-19 | 2 | 0 |
| 29-Dec-19 | 6 | 0 |
| 30-Dec-19 | 5 | 0 |
| 31-Dec-19 | 2 | 0 |
| 1-Jan-20 | 10 | 0 |
| 2-Jan-20 | 7 | 0 |
| 3-Jan-20 | 13 | 0 |
| 4-Jan-20 | 13 | 0 |
| 5-Jan-20 | 15 | 0 |
| 6-Jan-20 | 19 | 0 |
| 7-Jan-20 | 31 | 0 |
| 8-Jan-20 | 44 | 0 |
| 9-Jan-20 | 32 | 0 |
| 10-Jan-20 | 33 | 0 |
| 11-Jan-20 | 33 | 0 |
| 12-Jan-20 | 30 | 0 |
| 13-Jan-20 | 22 | 0 |
| 14-Jan-20 | 23 | 0 |
| 15-Jan-20 | 23 | 0 |
| 16-Jan-20 | 13 | 0 |
| 17-Jan-20 | 6 | 0 |
| 18-Jan-20 | 6 | 0 |
| 19-Jan-20 | 2 | 0 |
| 20-Jan-20 | 72 | 5 |
| 21-Jan-20 | 105 | 44 |
| 22-Jan-20 | 69 | 62 |
| 23-Jan-20 | 105 | 154 |
| 24-Jan-20 | 180 | 264 |
| 25-Jan-20 | 323 | 365 |
| 26-Jan-20 | 371 | 398 |
| 27-Jan-20 | 1291 | 480 |
| 28-Jan-20 | 840 | 619 |
| 29-Jan-20 | 1032 | 705 |
| 30-Jan-20 | 1220 | 762 |
| 31-Jan-20 | 1347 | 755 |
| 1-Feb-20 | 1921 | 669 |
| 2-Feb-20 | 2103 | 726 |
| 3-Feb-20 | 2345 | 890 |
| 4-Feb-20 | 3156 | 731 |
| 5-Feb-20 | 2987 | 707 |
| 6-Feb-20 | 2447 | 696 |
| 7-Feb-20 | 2841 | 558 |
| 8-Feb-20 | 2147 | 509 |
| 9-Feb-20 | 2618 | 444 |
| 10-Feb-20 | 2097 | 381 |
| 11-Feb-20 | 1638 | 377 |
| 12-Feb-20 | 1508 | 312 |
| 13-Feb-20 | 1728 | 267 |
| 14-Feb-20 | 1282 | 221 |
| 15-Feb-20 | 955 | 166 |
| 16-Feb-20 |  | 115 |
| 17-Feb-20 |  | 81 |
| 18-Feb-20 |  | 56 |
| 19-Feb-20 | 628 | 45 |
| 20-Feb-20 | 631 | 258 |
| 21-Feb-20 | 366 | 31 |
| 22-Feb-20 | 630 | 18 |
| 23-Feb-20 | 398 | 11 |
| 24-Feb-20 | 499 | 9 |
| 25-Feb-20 | 401 | 5 |
| 26-Feb-20 | 409 | 24 |
| 27-Feb-20 | 318 | 9 |
| 28-Feb-20 | 423 | 4 |
| 29-Feb-20 | 570 | 3 |
| 1-Mar-20 | 196 | 6 |
| 2-Mar-20 | 114 | 11 |
| 3-Mar-20 | 115 | 4 |
| 4-Mar-20 | 134 | 5 |
| 5-Mar-20 | 126 | 17 |
| 6-Mar-20 | 74 | 25 |
| 7-Mar-20 | 41 | 3 |
| 8-Mar-20 | 36 | 4 |

***** According to the Diagnosis and Treatment Plan for Novel Coronavirus Pneumonia (5th Edition), the number of laboratory-confirmed patients in Hubei was no longer issued separately by the Hubei Provincial Health Commission. Although the 6th edition of the plan issued on February 18 requires release of laboratory-confirmed patients separately, we cannot obtain this number in Hubei from February 16 to February 18.

**Reference**

1. Platt H. Sequential minimal optimization (ed. Platt H.) (Theia Lucina Gerhild, 2012).

2. Marino S., Hogue I. B., Ray C. J., Kirschner D. E. A methodology for performing global uncertainty and sensitivity analysis in systems biology. *J*. *Theor*. *Biol*. **254**, 178–196 (2008).

3. McKay M. D., Beckman R. J. & Conover W. J. A comparison of three methods for selecting values of input variables in the analysis of output from a computer code. *Technometrics*. **42**, 55-61 (2020).
